# Gigantol Inhibits Epithelial to Mesenchymal Process in Human Lung Cancer Cells

**DOI:** 10.1155/2016/4561674

**Published:** 2016-08-29

**Authors:** Thitita Unahabhokha, Pithi Chanvorachote, Boonchoo Sritularak, Jutarat Kitsongsermthon, Varisa Pongrakhananon

**Affiliations:** ^1^Cell-Based Drug and Health Product Development Research Unit, Faculty of Pharmaceutical Sciences, Chulalongkorn University, Bangkok 10330, Thailand; ^2^Department of Pharmacology and Physiology, Faculty of Pharmaceutical Sciences, Chulalongkorn University, Bangkok 10330, Thailand; ^3^Department of Pharmacognosy and Pharmaceutical Botany, Faculty of Pharmaceutical Sciences, Chulalongkorn University, Bangkok 10330, Thailand; ^4^Department of Pharmaceutics and Industrial Pharmacy, Faculty of Pharmaceutical Sciences, Chulalongkorn University, Bangkok 10330, Thailand

## Abstract

Lung cancer remains a leading public health problem as evidenced by its increasing death rate. The main cause of death in lung cancer patients is cancer metastasis. The metastatic behavior of lung cancer cells becomes enhanced when cancer cells undergo epithelial to mesenchymal transition (EMT). Gigantol, a bibenzyl compound extracted from the Thai orchid,* Dendrobium draconis*, has been shown to have promising therapeutic potential against cancer cells, which leads to the hypothesis that gigantol may be able to inhibit the fundamental EMT process in cancer cells. This study has demonstrated for the first time that gigantol possesses the ability to suppress EMT in non-small cell lung cancer H460 cells. Western blot analysis has revealed that gigantol attenuates the activity of ATP-dependent tyrosine kinase (AKT), thereby inhibiting the expression of the major EMT transcription factor, Slug, by both decreasing its transcription and increasing its degradation. The inhibitory effects of gigantol on EMT result in a decrease in the level of migration in H460 lung cancer cells. The results of this study emphasize the potential of gigantol for further development against lung cancer metastasis.

## 1. Introduction

Epithelial to mesenchymal transition (EMT) is the hallmark of cancer metastasis [1–3]. According to several reports, the survival rate of lung cancer patients is significantly diminished upon the diagnosis of cancer metastasis [4–6]. The development of a compound with the potential to attenuate the EMT process has been gaining interest in pharmaceutical research as a potential anticancer treatment. The change of cancer cells from epithelial to mesenchymal phenotypes facilitates the aggressiveness of the cancer. Several proteins have been identified as markers of the transdifferentiation process [7]. Decreases in E-cadherin, a transmembrane protein responsible for intercellular interactions, have been reported during the transition into the mesenchymal phenotype [7–9]. On the other hand, N-cadherin is a cell-cell adhesion molecule in mesenchymal cells. The EMT process is characterized by a shift in expression from E-cadherin to N-cadherin [10, 11]. Likewise, it has been claimed that Vimentin, another protein necessary for the motility of the mesenchymal cells, is increased during the EMT process [12]. These changes in molecular expression are mainly driven by the Snail family of transcription factors, in particular Slug [13, 14]. The Slug expression is further regulated through transcription and degradation pathways. *β*-catenin is responsible for interacting with the transcriptional factor Slug and promotes the production of Slug at the level of DNA. Conversely, its stability is controlled by GSK-3*β*, which causes ubiquitination and degradation of Slug [1–3, 15–17]. Recent evidence has suggested that AKT is able to influence the activity of both *β*-catenin and GSK-3*β* [4–6, 12, 15, 17]. An increase in AKT activity is also associated with EMT incidence in cancer cells [7, 18]. Taken together, these molecular pathways have an important role in the induction of cancer cells towards EMT. Alterations of the activity or expression of these molecules could potentially prevent cancer metastasis.

**Figure 1:**
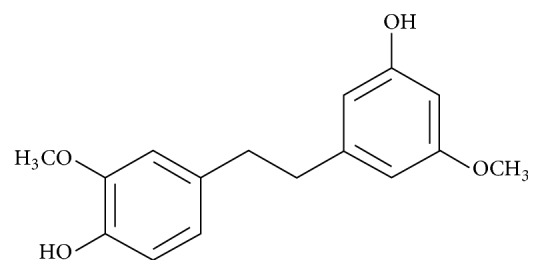
Chemical structure of gigantol.

Several pure compound extractions from Thai medicinal orchids, including gigantol, have been reported to have promising anticancer activity [18–23]. Gigantol (Figure 1) is a stilbenoid derivative isolated from the stem of* Dendrobium draconis*, a Thai medicinal orchid [10, 11, 24]. The anticancer activity of gigantol has been widely reported [12, 18–20]; however, its effect on EMT inhibition and the underlying mechanisms have yet to be clarified. It is possible that gigantol may have the potential to attenuate the regulatory mechanisms of EMT, leading to a decrease in aggressive cancer cell behavior. This result would support the development of this compound for cancer therapy.

## 2. Materials and Methods

### 2.1. Materials

Gigantol was isolated from* Dendrobium draconis* as previously described [23]. Gigantol used in this study was isolated from the dried powdered stems of* D. draconis* and extracted by MeOH using vacuum-liquid chromatography (VLC) and column chromatography (CC) with more than 95% purity. Gigantol was prepared in dimethyl sulfoxide (DMSO) for stock solution and PBS (phosphate-buffered saline) was used to dilute the stock solution into working concentrations. The final concentration of DMSO used in all of the experiments was 0.1%. The results from the treatment groups were compared with the untreated control exposed to the 0.1% final concentration of DMSO. DMSO, 3-(4,5-dimethylthiazol-2-yl)-2,5-diphenyltetrazolium bromide (MTT), Hoechst 33342, propidium iodide (PI), bovine serum albumin (BSA), and antibody for ubiquitin were purchased from Sigma Chemical, Inc. (St. Louis, MO, USA). Antibodies for Rho GTP and Rac GTP were purchased from NewEast Bioscience (King of Prussia, PA, USA). Antibodies for N-cadherin, E-cadherin, Vimentin, Snail, ZEB-1, Slug, *β*-catenin, phosphorylated AKT (Ser473), AKT, phosphorylated GSK-3*β* (Ser9), GSK-3*β*, GAPDH, and peroxidase-conjugated secondary antibodies were purchased from Cell Signaling (Danvers, MA, USA).

### 2.2. Cell Culture

Human non-small cell lung cancer cells (NCI-H460) were obtained from the American Type Culture Collection (ATCC, Manassas, VA, USA) and cultured in Roswell Park Memorial Institute (RPMI) 1640 medium supplemented with 10% fetal bovine serum (FBS), 2 mM L-glutamine, 100 U/mL penicillin, and 100 *μ*g/mL streptomycin. Cells were cultured at 37°C in a humidified incubator with 5% CO_2_ and passaged at near confluence with trypsin-EDTA. RPMI 1640 medium, FBS, L-glutamine, penicillin, streptomycin, PBS, trypsin, and EDTA were purchased from GIBCO (Grand Island, NY, USA).

### 2.3. Cytotoxic Assay

Cell viability was examined using a colorimetric 3-(4,5-dimethylthiazol-2-yl)-2,5-diphenyltetrazolium bromide (MTT) assay. H460 cells were seeded in 96-well plates at 10,000 cells/well and incubated overnight at 37°C. After being exposed to gigantol treatments at various doses (0–50 *μ*M) for 24 h, the medium was removed and 100 *μ*L of MTT solution was added to each well. Then the plates were further incubated for 4 h at 37°C. After that the medium was replaced by 100 *μ*L of DMSO to dissolve the formazan crystal. The intensity of formazan produce was measured at 570 nm using a microplate reader (Anthros, Durham, NC, USA). Cell viability was presented as percentage from the absorbance of the treatment groups in relative to the control group.

### 2.4. Apoptosis Assay

Apoptotic and necrotic cells were identified using a fluorescent nuclear staining dye, Hoechst 33342, and PI. H460 cells were seeded in 96-well plates at 10,000 cells/well and incubated overnight at 37°C. After exposing to gigantol treatments at various doses (0–50 *μ*M) for 24 h, cells were washed and incubated with 10 *μ*g/mL Hoechst 33342 and 5 *μ*g/mL PI for 30 min in the dark. Nuclei condensation and DNA fragmentation of apoptotic and necrotic cells were observed and scored using fluorescence microcopy (Olympus IX51 with DP70). The data were presented as percentage from the number of apoptotic and necrotic cells of the treatment groups relative to the control group.

### 2.5. Migration Assay

Cell migration was observed using wound healing and transwell migration assay. H460 cells were treated with gigantol at noncytotoxic concentrations for 24 h before subjecting to migration evaluation. For wound healing assay, treated H460 cells were seeded in 24-well plates at 250,000 cells/well and incubated overnight at 37°C. After monolayer of cells was formed, a micropipette tip was used to create a wound space. Then the cell debris was removed by washing with PBS and replaced with serum-free culture medium. The cell migration level across the wounded space was observed and evaluated using inverted microscope (Olympus IX51 with DP70). The relative migration level was calculated from the difference of the wound space between the treatment group and the control group divided by the wound space of the control group at each evaluation time. For transwell migration assay, the treated H460 cells were seeded at 25,000 cells/well into the upper chamber of 24-transwell plates in a serum-free culture medium while culture medium with 10% FBS was added to the lower chamber. After incubating at 37°C for 24 h, the leftover cells on the upper chamber were removed and the migrated cells in the lower chamber were stained with 10 *μ*g/mL Hoechst 33342 for 30 min in the dark. The Hoechst staining cells were photographed and analyzed using fluorescence microcopy (Olympus IX51 with DP70).

### 2.6. Invasion Assay

Cell invasion was evaluated using transwell invasion assay. H460 cells were treated with gigantol at noncytotoxic concentrations for 24 h before subjecting to invasion evaluation. Before the experiment, 0.5% of matrigel was coated on the filter membrane of the transwell chamber and left overnight at 37°C. Then, the treated H460 cells were seeded at 25,000 cells/well into the upper chamber of 24-transwell plates in a serum-free culture medium while culture medium with 10% FBS was added to the lower chamber. After incubating at 37°C for 24 h, the noninvaded cells in the upper chamber were removed and the invaded cells in the lower chamber were stained with 10 *μ*g/mL Hoechst 33342 for 30 min in the dark. The Hoechst staining cells were photographed and analyzed using fluorescence microcopy (Olympus IX51 with DP70).

### 2.7. Western Blot Analysis

Levels of protein expression were evaluated using Western blot analysis. H460 cells were exposed to treatment of gigantol at noncytotoxic concentrations. After specific treatments, cells were harvested by washing twice with cold PBS and incubated with lysis buffer containing 20 mM Tris-HCl (pH 7.5), 1% Triton X-100, 150 mM sodium chloride, 10% glycerol, 1 mM sodium orthovanadate, 50 mM sodium fluoride, 100 mM phenylmethylsulfonyl fluoride, and protease inhibitor cocktail for 1 h at 4°C. The cell lysate was collected as protein sample and subjected to protein concentration measurement using the BCA assay kit (Bio-Rad Laboratories, Hercules, CA, USA). Protein from each sample was denatured by heating at 95°C for 5 min with Laemmli loading buffer prior to the gel electrophoresis. Then protein samples were separated by molecular weight using precast 5–10% gradient SDS-PAGE gel and transferred to nitrocellulose membranes. After blocking with 5% skim milk for 1 h, the membranes were incubated with the indicated primary antibodies at 4°C overnight. After that, the membranes were washed thoroughly with TBST (25 mM Tris-HCl (pH 7.5), 125 mM NaCl, and 0.05% Tween 20), and then they were incubated with horseradish peroxide-conjugated secondary antibodies for an additional hour at room temperature. Subsequently, the bands were then visualized using a film exposure with a chemiluminescence detection system and quantified using analyst/PC densitometry software by Image J.

### 2.8. Immunoprecipitation Assay

Levels of protein interaction were evaluated using immunoprecipitation assay. In order to detect the ubiquitin-protein complex, lactacystin was pretreated to the cells an hour prior to gigantol treatment. After specific treatments, cells were harvested and lysed with lysis buffer. Then the cell lysate was separated and collected by centrifuging at 12,000 rpm for 3 min at 4°C before preclearing with agarose bead for 45 min at 4°C to prevent unspecific binding. The remaining cell lysate was subjected to protein measurement for equal loading. Next the anti-Slug antibody was added to the cleared lysate and incubated overnight at 4°C before further adding agarose beads for an additional 2 h at 4°C. The precipitated immune complexes were washed with ice-cold lysis buffer, resuspended in 2x Laemmli sample buffer, and then heated at 95°C for 5 min. After that, immune complexes were separated using precast 5–10% gradient SDS-PAGE gel electrophoresis and transferred to nitrocellulose membranes; the Western blot analysis was then performed using an anti-ubiquitin antibody.

### 2.9. Statistical Analysis

Results were expressed as mean ± standard error (SE) from at least four independently performed experiments. Differences between treatments were examined using the one-way analysis of variance (ANOVA) followed by* post hoc* test. *p* values less than 0.05 were considered statistically significant.

## 3. Results

### 3.1. Cytotoxicity of Gigantol on Lung Cancer H460 Cells

**Figure 2:**
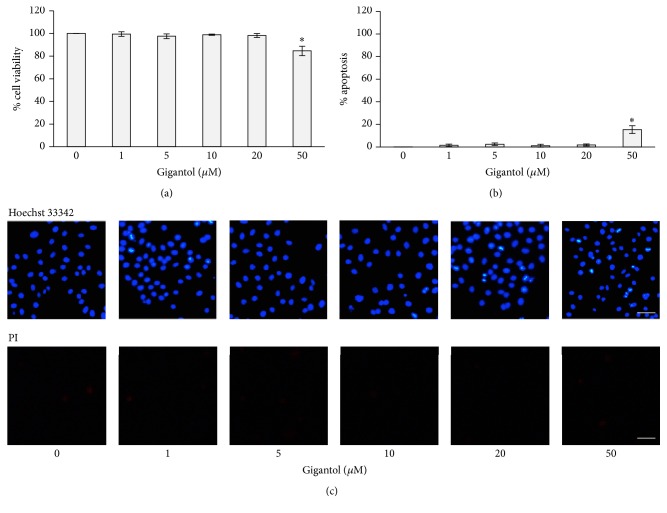
Effect of gigantol on human lung cancer cell H460 cytotoxicity. (a) H460 cells were treated with various concentrations (0–50 *μ*M) of gigantol for 24 h, and cell viability was measured by the MTT assay. The viability of untreated control cells was represented as 100%. (b) H460 cells were treated with various concentrations (0–50 *μ*M) of gigantol for 24 h, and apoptotic cell death was evaluated using Hoechst 33342 nuclear staining dye. The percentages of cells undergoing apoptosis were calculated comparing to the untreated control cells. (c) The fluorescence images were captured after staining with either Hoechst 33342 or propidium iodide (PI) (*scale bar* is 50 *μ*m). The data represent mean ± SE (*n* = 4). ^*∗*^ *p* < 0.05 versus untreated control cells.

To determine the concentration of gigantol used in this study, we first evaluated the cytotoxicity of gigantol using MTT and apoptosis assays. Before cell viability evaluation, H460 cells were treated with various concentrations of gigantol (0–50 *μ*M) for 24 h.  Figure 2(a) illustrates that, at concentrations lower than 50 *μ*M, there was no significant impact of gigantol on cell viability. The apoptosis assay confirmed that low concentrations of gigantol (0, 1, 5, 10, or 20 *μ*M) could not induce apoptosis in cells (Figure 2(b)). Approximately 15% of the cells treated with 50 *μ*M of gigantol showed signs of nuclear condensation, an indicator of apoptosis (Figure 2(c)). Noncytotoxic concentrations of gigantol (0–20 *μ*M) were used in subsequent experiments.

### 3.2. Gigantol Suppresses Epithelial to Mesenchymal Transition (EMT) in Lung Cancer H460 Cells

**Figure 3:**
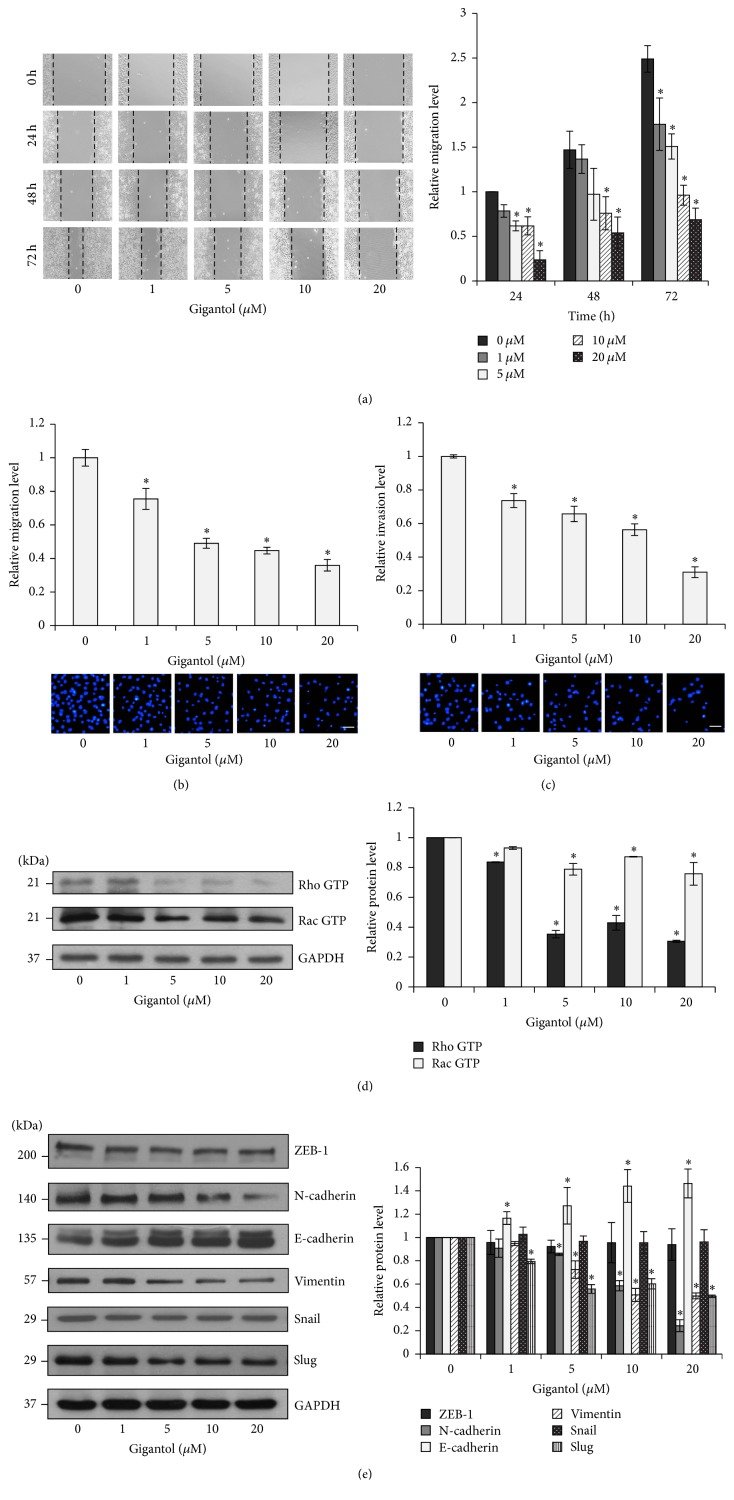
Effect of gigantol on epithelial to mesenchymal process (EMT) in human lung cancer cell H460. (a) H460 cells were treated with noncytotoxic doses of gigantol (0–20 *μ*M) for 24 h. Wound space was photographed and analyzed at 0, 24, 48, and 72 h. The relative migration level was calculated as the changes of wound space of the treatment groups compared to that of the untreated control group at the indicated time. (b) H460 cells migration was examined using transwell migration assay. After 24 h the migrated cells were stained with Hoechst 33342 and visualized by fluorescence microscopy (*scale bar* is 50 *μ*m). The relative migration level was calculated as the number of migrated cells of the treatment groups divided by that of the untreated control group. (c) H460 cells invasion was examined using transwell invasion assay. After 24 h the invaded cells were stained with Hoechst 33342 and visualized by fluorescence microscopy (*scale bar* is 50 *μ*m). The relative invasion level was calculated as the number of migrated cells of the treatment groups divided by that of the untreated control group. (d) The effect of gigantol on migratory-related proteins. After H460 cells were treated with noncytotoxic doses of gigantol (0–20 *μ*M) for 24 h, the expression of Rho GTP and Rac GTP was evaluated using Western blot assay. (e) The effect of gigantol on EMT marker proteins. After H460 cells were treated with noncytotoxic doses of gigantol (0–20 *μ*M) for 24 h, the expressions of N-cadherin, E-cadherin, Vimentin, Snail, Slug, and ZEB-1 were evaluated using Western blot assay. The blots were reprobed with GAPDH to confirm equal loading. The immunoblot signals were qualified by densitometry. The data represent mean ± SE (*n* = 4). ^*∗*^ *p* < 0.05 versus untreated control cells.

Cellular migration is an indicator of cells undergoing EMT; therefore, the migration levels of H460 were examined to determine the effect of gigantol on EMT inhibition. The H460 cells were pretreated with gigantol at 1, 5, 10, and 20 *μ*M for 24 h, and then the migration level was assessed using wound healing and transwell migration assays.  Figure 3(a) shows that treatment with 20 *μ*M gigantol suppressed cell migration across the wound space (approximately 70%) at intervals as early as 24 h. At 72 h, gigantol concentrations of 1, 5, 10, or 20 *μ*M were able to significantly attenuate H460 cell motility when compared to the control. Consistently, results from the transwell migration assay demonstrated that gigantol was able to decrease the number of cells moving across the transwell filter within 24 h in a dose-dependent manner (Figure 3(b)). A similar trend was also observed in a transwell invasion assay. Approximately 30%, 35%, 45%, and 70% reductions in invasion were recorded in H460 cells treated for 24 h with concentrations of 1, 5, 10, and 20 *μ*M of gigantol, respectively (Figure 3(c)). To evaluate migration activity at the molecular level, the expression levels of Rho GTP and Rac GTP in H460 cells were evaluated after 24 h of treatment with gigantol. Rho GTP is responsible for stress fiber extension, while Rac GTP regulates lamellipodia formation [25–27]. Both proteins are known migration regulator proteins that enhance cytoskeleton reorganization by facilitating membrane protrusion at the edges of the cell. The results from the Western blot analysis shown in Figure 3(d) demonstrate that treatment of gigantol suppressed Rho GTP and Rac GTP expression. To determine the existence of an association between gigantol and EMT, the expression of EMT marker proteins including E-cadherin, N-cadherin, Vimentin, Snail, Slug, and ZEB-1 was evaluated. The results from the Western blot analysis support our hypothesis that treatment with various concentrations of gigantol (0–20 *μ*M) for 24 h switched the cadherin type from E-cadherin to N-cadherin in the treated H460 cells (Figure 3(e)). In addition, 1, 5, 10, and 20 *μ*M of gigantol reduced Vimentin expression levels by 90%, 70% 50%, and 40%, respectively. Gigantol treatment also suppressed the expression levels of the Slug transcription factor, while Snail and ZEB-1 levels remained unchanged. This result suggests that gigantol is able to inhibit the EMT process by decreasing the production of the EMT transcription factor Slug.

### 3.3. Gigantol Increases Ubiquitination of the Slug Transcription Factor

**Figure 4:**
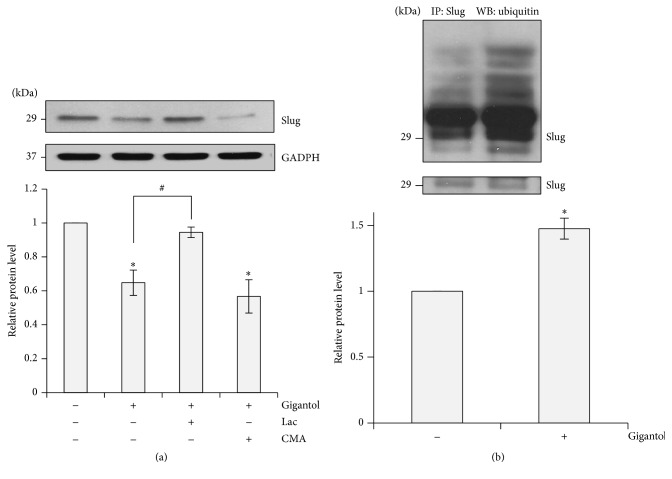
The effect of gigantol on Slug degradation process. (a) H460 cells were pretreated with a proteasomal inhibitor lactacystin (Lac) 10 *μ*M or lysosomal inhibitor concanamycin A (CMA) 1 *μ*M for an hour before treatment with 20 *μ*M of gigantol for 24 h. Slug expression was analyzed using Western blotting assay. The immunoblot signals were qualified by densitometry. The data represent mean ± SE (*n* = 4). ^*∗*^ *p* < 0.05 versus untreated control cells ^#^ *p* < 0.05 versus gigantol treated cells. (b) H460 cells were pretreated with lactacystin (Lac) 10 *μ*M for an hour, and then the pretreated cells were exposed to a presence of gigantol or left untreated for 3 h. The levels of immunocomplexes were analyzed for ubiquitin using anti-ubiquitin antibody. The blot was reprobed with Slug antibody to confirm the equal basal amount of Slug. Immunoblot signals were qualified by densitometry. The data represent mean ± SE (*n* = 4). ^*∗*^ *p* < 0.05 versus control cells.

We have demonstrated that gigantol is able to downregulate Slug expression. The objective of this experiment was to further investigate the mechanism by which gigantol treatment downregulates Slug expression. It was reported that the stability of Slug is controlled by proteasomal degradation [12, 28]. Protein degradation occurs either via proteasomal or via lysosomal pathways. To determine which pathway contributes to Slug downregulation, H460 cells were treated with either the proteasomal inhibitor lactacystin (Lac) or the lysosomal inhibitor concanamycin A (CMA).  Figure 4(a) shows that Lac was able to inhibit the reduction of Slug in response to gigantol. This indicated that Slug degradation was blocked in proteasomal-suppressed cells resulting in an increased accumulation of Slug in the treated cells when compared to the control. In contrast, CMA treatment had no effect on Slug expression levels. This finding reveals that proteasomal degradation is involved in the stability of Slug expression. It is known that ubiquitination is a critical prerequisite and a rate-limiting step prior to proteasomal cleavage. Because of this fact, we investigated Slug-ubiquitin complexes in response to gigantol treatment using an immunoprecipitation assay.  Figure 4(b) shows that the H460 cells treated with gigantol exhibited significant increases in Slug-ubiquitin complex levels, despite the equally loaded Slug expression in the control and the treatment groups. This suggests that gigantol is able to enhance Slug degradation via the proteasomal pathway.

### 3.4. Effect of Gigantol on EMT Regulating Proteins

**Figure 5:**
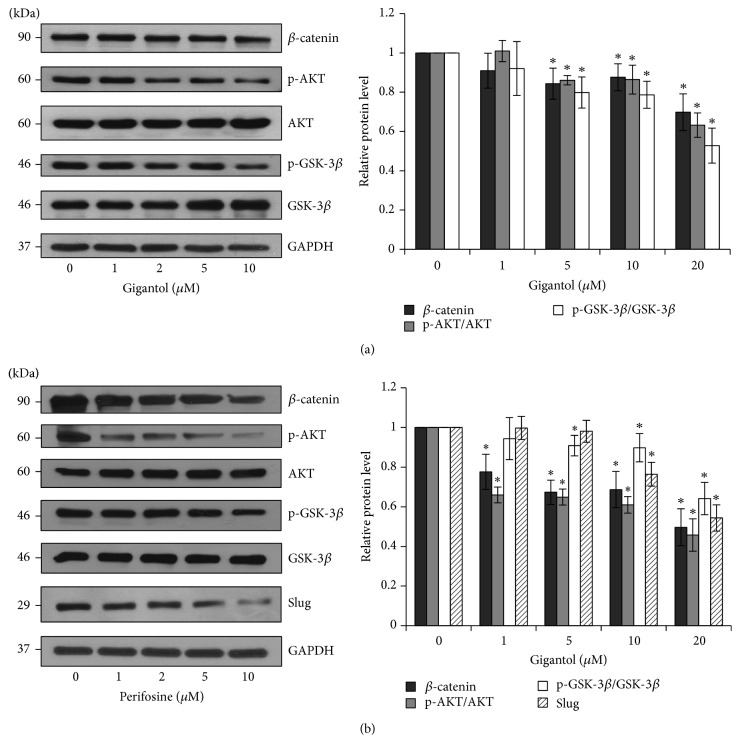
The effect of gigantol on EMT regulating proteins. (a) After H460 cells were treated with noncytotoxic doses of gigantol (0–20 *μ*M), the expression of *β*-catenin, phosphorylated AKT (Ser473), AKT, phosphorylated GSK-3*β* (Ser9), and GSK-3*β* was evaluated using Western blot assay. (b) AKT inhibitor, perifosine, was used to treat H460 cells (0–10 *μ*M); the expression of *β*-catenin, phosphorylated AKT (Ser473), AKT, phosphorylated GSK-3*β* (Ser9), GSK-3*β*, and Slug was evaluated using Western blot assay. The blots were reprobed with GAPDH to confirm equal loading. The immunoblot signals were qualified by densitometry. The data represent mean ± SE (*n* = 4). ^*∗*^ *p* < 0.05 versus untreated control cells.

To further examine the signaling pathway of gigantol in inhibiting the EMT process, the expression levels of the upstream proteins were evaluated. It has been shown that GSK-3*β* is the protein responsible for Slug ubiquitination.  Figure 5(a) shows that gigantol treatment decreased inactivated GSK-3*β* (p-GSK-3*β*) levels, indicating that GSK-3*β* Slug destabilization was increased. Moreover, *β*-catenin is known as an essential transcriptional activator of Slug expression [10]. Interestingly, Figure 5(a) clearly shows that treatment with gigantol could decrease *β*-catenin expression. Moreover, previous studies have indicated that AKT enhancement drives epithelial cells towards transdifferentiation [10]. The data shown in Figure 5(a) demonstrate that AKT phosphorylation was significantly reduced in response to gigantol treatment. These results suggest that gigantol attenuates EMT via AKT downregulation, leading to a decrease in *β*-catenin expression and an increase in GSK-3*β* activity.

To further confirm that the inhibition of AKT phosphorylation is able to interfere with downstream proteins, H460 cells were treated with the AKT inhibitor, perifosine. The data shown in Figure 5(b) confirm that perifosine treatment suppressed AKT phosphorylation. In addition, the *β*-catenin and p-GSK-3*β* expression levels were significantly reduced. These results confirm that AKT activation could influence the expression of downstream proteins and the EMT process.

## 4. Discussion

Cancer metastasis is a fundamental cause of death in lung cancer patients. The essential driving force towards metastasis is the morphological transition of cells known as epithelial to mesenchymal transition [3]. This transition in cellular phenotype facilitates the aggressiveness of cancer by enhancing cellular migration levels and anoikis resistance. Several studies have identified various natural compounds with the ability to attenuate cancer metastasis. It has been reported that gigantol, a stilbenoid derivative extracted from* Dendrobium draconis*, possesses promising anticancer properties [18–20, 28]. In this study, we have provided further molecular evidence supporting the potential of gigantol as a biological agent for cancer treatment. Our results demonstrate that noncytotoxic concentrations of gigantol are able to significantly inhibit migratory behavior and decrease the level of EMT marker proteins (Figure 3). Moreover, our findings indicate that these inhibitory effects are involved in gigantol downregulation of Slug, a major transcription factor underlying EMT [12, 13].

Previous studies have reported that gigantol inhibits migration and sensitizes anoikis in lung cancer cells [18, 28]. However, scientific evidence on the underlying mechanisms of upstream pathways remained unknown. Consistent with a previous report [18], our results demonstrate that gigantol inhibits the ability of lung cancer cells to migrate. Interestingly, in the present study, gigantol pretreatment occurred 24 h prior to the migration evaluation, and we found that the inhibition of migration persisted up to 72 h after gigantol treatment was removed (Figure 3). This finding provides novel evidence that gigantol possesses the ability to affect upstream mechanisms of migration.

It is widely accepted that cellular migration and anoikis resistance are properties of the EMT process [7, 29–31]. We therefore hypothesized that gigantol may attenuate the transdifferentiation process. Our protein expression analysis demonstrated that gigantol was able to significantly reduce the expression of EMT markers including N-cadherin and Vimentin while enhancing the expression of E-cadherin 24 h after treatment (Figure 3). Slug, the main regulator of EMT, acts as a molecular switch that suppresses E-cadherin expression by blocking a set of E-cadherin encoding genes. Our results show that gigantol treatment significantly suppressed the expression of the Slug transcription factor. It was also observed that gigantol did not affect Snail or ZEB-1 expression. It appears that Slug is the main target of gigantol treatment. Another possibility is that Snail was claimed to be an unstable protein. So the effect of gigantol on the expression of Slug may be detectable but not Snail or ZEB-1. This finding suggests that gigantol was able to attenuate the EMT process at the transcriptional level. *β*-catenin and GSK-3*β* proteins may regulate the expression of the Slug transcription factor via production and degradation pathways, respectively [12]. In epithelial cells, *β*-catenin interacts with the cytoplasmic domain of E-cadherin, whereas, during the EMT process, *β*-catenin is released from the complex and translocated into the nucleus to increase the expression of Slug. On the other hand, activated GSK-3*β* causes Slug phosphorylation in the central domain, leading to Slug ubiquitination and, consequently, proteasomal degradation [12]. GSK-3*β* can be inactivated through phosphorylation. The results illustrated in Figure 5(a) indicate that gigantol treatment not only decreased Slug expression by promoting the degradation pathway but also suppressed its transcriptional activation.

Many studies have demonstrated that activated AKT plays an important role in the EMT process [10, 32]. As evidenced by a previous report, gigantol inhibited migration by decreasing the function of AKT [18]. However, in the present study, it was shown that AKT activation was significantly suppressed within the first 3 h of gigantol treatment (Figure 5(a)). Therefore, it is possible that gigantol may affect the upstream signaling pathway to attenuate migration behavior. These data are consistent with previous studies that show that the attenuation of AKT activity was able to suppress the expression of Slug and inhibit mesenchymal transition through the *β*-catenin and GSK-3*β* pathways [32–34]. It has been demonstrated that the activation of AKT positively regulates Slug transcription via *β*-catenin* in vitro* [35] and suppresses Slug degradation [36]. The inhibition of AKT could be a promising therapeutic approach to attenuate the EMT process.

## 5. Conclusion

**Figure 6:**
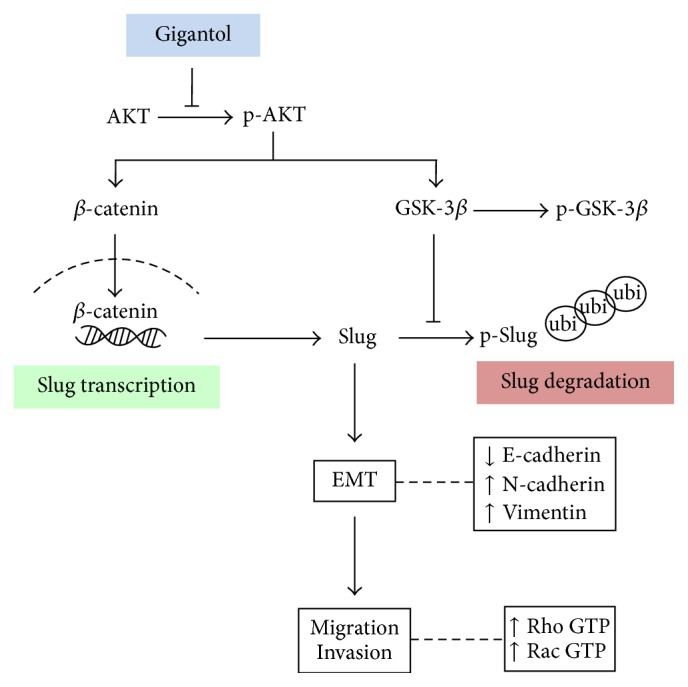
A schematic diagram summarizes the EMT inhibitory mechanism of gigantol on lung cancer cells. Gigantol suppresses the activation of AKT resulting in a decrease in Slug by both decreasing the production and increasing the degradation processes.

In conclusion, this study has demonstrated that gigantol is able to attenuate the EMT process in lung cancer cells. The reduction of AKT activity decreased the transcription and the stability of Slug. Gigantol was shown to reduce *β*-catenin activity and Slug transcription while enhancing GSK-3*β* ubiquitination of Slug, resulting in decreased Slug levels and thereby suppressing the EMT process (Figure 6). This novel discovery supports the future development of gigantol as an antimetastasis treatment in cancer patients.

## References

[1] Kong D., Li Y., Wang Z., Sarkar F. H. (2011). Cancer stem cells and Epithelial-to-Mesenchymal Transition (EMT)-phenotypic cells: are they cousins or twins?. *Cancers*.

[2] Chen X., Zhang J., Zhang Z., Li H., Cheng W., Liu J. (2013). Cancer stem cells, epithelial-mesenchymal transition, and drug resistance in high-grade ovarian serous carcinoma. *Human Pathology*.

[3] Geiger T. R., Peeper D. S. (2009). Metastasis mechanisms. *Biochimica et Biophysica Acta (BBA)—Reviews on Cancer*.

[4] American Cancer Society (2016). *Cancer Facts & Figures*.

[5] Pasquier J., Abu-Kaoud N., Thani A. I., Rafii A. (2015). Epithelial to mesenchymal transition in a clinical perspective. *Journal of Oncology*.

[6] Iwatsuki M., Mimori K., Yokobori T. (2010). Epithelial-mesenchymal transition in cancer development and its clinical significance. *Cancer Science*.

[7] Voulgari A., Pintzas A. (2009). Epithelial-mesenchymal transition in cancer metastasis: mechanisms, markers and strategies to overcome drug resistance in the clinic. *Biochimica et Biophysica Acta*.

[8] Kumar S., Park S. H., Cieply B. (2011). A pathway for the control of anoikis sensitivity by E-cadherin and epithelial-to-mesenchymal transition. *Molecular and Cellular Biology*.

[9] Shi Y., Wu H., Zhang M., Ding L., Meng F., Fan X. (2013). Expression of the epithelial-mesenchymal transition-related proteins and their clinical significance in lung adenocarcinoma. *Diagnostic Pathology*.

[10] Lamouille S., Xu J., Derynck R. (2014). Molecular mechanisms of epithelial-mesenchymal transition. *Nature Reviews Molecular Cell Biology*.

[11] Wheelock M. J., Shintani Y., Maeda M., Fukumoto Y., Johnson K. R. (2008). Cadherin switching. *Journal of Cell Science*.

[12] Lee K., Nelson C. M. (2012). Insights into the regulation of epithelial-mesenchymal transition and tissue fibrosis. *The Journal of Cell Biology*.

[13] Barrallo-Gimeno A., Nieto M. A. (2005). The Snail genes as inducers of cell movement and survival: implications in development and cancer. *Development*.

[14] Frisch S. M., Schaller M., Cieply B. (2013). Mechanisms that link the oncogenic epithelial-mesenchymal transition to suppression of anoikis. *Journal of Cell Science*.

[15] Sánchez-Tilló E., Liu Y., De Barrios O. (2012). EMT-activating transcription factors in cancer: beyond EMT and tumor invasiveness. *Cellular and Molecular Life Sciences*.

[16] Wang Y., Shi J., Chai K., Ying X., Zhou B. P. (2013). The role of Snail in EMT and tumorigenesis. *Current Cancer Drug Targets*.

[17] Shih J.-Y., Yang P.-C. (2011). The EMT regulator slug and lung carcinogenesis. *Carcinogenesis*.

[18] Charoenrungruang S., Chanvorachote P., Sritularak B., Pongrakhananon V. (2014). Gigantol, a bibenzyl from *Dendrobium draconis*, inhibits the migratory behavior of non-small cell lung cancer cells. *Journal of Natural Products*.

[19] Charoenrungruang S., Chanvorachote P., Sritularak B. (2014). Gigantol-induced apoptosis in lung cancer cell through mitochondrial-dependent pathway. *Thai Journal of Pharmaceutical Sciences*.

[20] Bhummaphan N., Chanvorachote P. (2015). Gigantol suppresses cancer stem cell-like phenotypes in lung cancer cells. *Evidence-Based Complementary and Alternative Medicine*.

[21] Ho C.-K., Chen C.-C. (2003). Moscatilin from the orchid *Dendrobrium loddigesii* is a potential anticancer agent. *Cancer Investigation*.

[22] Klongkumnuankarn P., Busaranon K., Chanvorachote P., Sritularak B., Jongbunprasert V., Likhitwitayawuid K. (2015). Cytotoxic and antimigratory activities of phenolic compounds from *Dendrobium brymerianum*. *Evidence-Based Complementary and Alternative Medicine*.

[23] Sritularak B., Anuwat M., Likhitwitayawuid K. (2011). A new phenanthrenequinone from *Dendrobium draconis*. *Journal of Asian Natural Products Research*.

[24] Price L. S., Leng J., Schwartz M. A., Bokoch G. M. (1998). Activation of Rac and Cdc42 by integrins mediates cell spreading. *Molecular Biology of the Cell*.

[25] Van Aelst L., Symons M. (2002). Role of Rho family GTPases in epithelial morphogenesis. *Genes & Development*.

[26] Zhao X., Guan J.-L. (2011). Focal adhesion kinase and its signaling pathways in cell migration and angiogenesis. *Advanced Drug Delivery Reviews*.

[27] Wu Y., Evers B. M., Zhou B. P. (2009). Small C-terminal domain phosphatase enhances snail activity through dephosphorylation. *The Journal of Biological Chemistry*.

[28] Unahabhokha T., Chanvorachote P., Pongrakhananon V. (2016). The attenuation of epithelial to mesenchymal transition and induction of anoikis by gigantol in human lung cancer H460 cells. *Tumor Biology*.

[29] Chiarugi P., Giannoni E. (2008). Anoikis: a necessary death program for anchorage-dependent cells. *Biochemical Pharmacology*.

[30] Nurwidya F., Takahashi F., Murakami A., Takahashi K. (2012). Epithelial mesenchymal transition in drug resistance and metastasis of lung cancer. *Cancer Research and Treatment*.

[31] Fenouille N., Tichet M., Dufies M. (2012). The epithelial-mesenchymal transition (EMT) regulatory factor SLUG (SNAI2) is a downstream target of SPARC and AKT in promoting melanoma cell invasion. *PLoS ONE*.

[32] Kiratipaiboon C., Tengamnuay P., Chanvorachote P. (2016). Ciprofloxacin improves the stemness of human dermal papilla cells. *Stem Cells International*.

[33] Zhao S., Fu J., Liu X., Wang T., Zhang J., Zhao Y. (2012). Activation of Akt/GSK-3beta/beta-catenin signaling pathway is involved in survival of neurons after traumatic brain injury in rats. *Neurological Research*.

[34] Fang D., Hawke D., Zheng Y. (2007). Phosphorylation of *β*-catenin by AKT promotes *β*-catenin transcriptional activity. *The Journal of Biological Chemistry*.

[35] Huang T.-S., Li L., Moalim-Nour L. (2015). A regulatory network involving *β*-catenin, e-cadherin, PI3k/Akt, and slug balances self-renewal and differentiation of human pluripotent stem cells in response to wnt signaling. *STEM CELLS*.

[36] Kao S.-H., Wang W.-L., Chen C.-Y. (2014). GSK3*β* controls epithelial-mesenchymal transition and tumor metastasis by CHIP-mediated degradation of slug. *Oncogene*.

